# Systematic Age-Related Differences in Chronic Disease Management in a Population-Based Cohort Study: A New Paradigm of Primary Care Is Required

**DOI:** 10.1371/journal.pone.0091340

**Published:** 2014-03-14

**Authors:** Alessandra Buja, Gianfranco Damiani, Rosa Gini, Modesta Visca, Bruno Federico, Daniele Donato, Paolo Francesconi, Alessandro Marini, Andrea Donatini, Salvatore Brugaletta, Vincenzo Baldo, Maria Donata Bellentani

**Affiliations:** 1 Laboratory of Public Health and Population Studies, Section Public Health, Dipartimento di Medicina Molecolare, Università di Padova, Padova, Italy; 2 Istituto di Sanità pubblica, Facoltà di Medicina, Università Cattolica Sacro Cuore di Roma, Roma, Italy; 3 Osservatorio di Epidemiologia, Agenzia Regionale di Sanità della Toscana, Firenze, Italy; 4 Sezione OSS - Organizzazione Servizi Sanitari, Agenas, Agenzia Nazionale per i Servizi Sanitari, Roma, Italy; 5 Dipartimento di Scienze Umane, Sociali e della Salute, Università degli Studi di Cassino e del Lazio Meridionale, Cassino, Italy; 6 ULSS 16 Padova, Regione Veneto, Padova, Italy; 7 Zona Territoriale Senigallia, Regione Marche, Ancona, Italy; 8 Regione Emilia Romagna, Bologna, Italy; 9 ASP 7 Ragusa, Regione Sicilia, Ragusa, Italy; 10 Laboratory of Public Health and Population Studies, Dipartimento di Medicina Molecolare, Università di Padova, Padova, Italy; RAND Corporation, United States of America

## Abstract

**Background:**

Our interest in chronic conditions is due to the fact that, worldwide, chronic diseases have overtaken infectious diseases as the leading cause of death and disability, so their management represents an important challenge for health systems. The aim of this study was to compare the performance of primary health care services in managing diabetes, congestive heart failure (CHF) and coronary heart disease (CHD), by age group.

**Methods:**

This population-based retrospective cohort study was conducted in Italy, enrolling 1,948,622 residents ≥16 years old. A multilevel regression model was applied to analyze compliance to care processes with explanatory variables at both patient and district level, using age group as an independent variable, and adjusting for sex, citizenship, disease duration, and Charlson index on the first level, and for District Health Unit on the second level.

**Results:**

The quality of chronic disease management showed an inverted U-shaped relationship with age. In particular, our findings indicate lower levels for young adults (16–44 year-olds), adults (45–64), and oldest old (+85) than for patients aged 65–74 in almost all quality indicators of CHD, CHF and diabetes management. Young adults (16–44 y), adults (45–64 y), the very old (75–84 y) and the oldest old (+85 y) patients with CHD, CHF and diabetes are less likely than 65–74 year-old patients to be monitored and treated using evidence-based therapies, with the exceptions of echocardiographic monitoring for CHF in young adult patients, and renal monitoring for CHF and diabetes in the very old.

**Conclusion:**

Our study shows that more effort is needed to ensure that primary health care systems are sensitive to chronic conditions in the young and in the very elderly.

## Background

The rising prevalence of chronic diseases all over the world is a growing cause of concern in the public health sector. Worldwide, chronic diseases have overtaken infectious diseases as the leading cause of death and disability. Non-communicable diseases now account for 63% of the world’s annual deaths, and approximately half of the global burden of disease [1]. Efforts have been made to identify strategies to prevent or reduce the risk of chronic diseases, as well as to organize an appropriate secondary prevention and management of chronic diseases to reduce the associated complications. There is currently a growing interest in the developed countries in redesigning health care organizations [2], focusing on practices to improve the quality of care and guarantee an equitable, timely and effective management of chronic diseases [3].

Disease management programs include measuring processes and outcomes [4]. Carefully designed, evidence-based care processes, sustained by automated clinical information and decision support systems, offer the highest chances of achieving the best outcomes from care provided for chronic conditions [5]. Clinical process indicators for assessing the medical management of chronic diseases are widely used for the purposes of producing evidence of the quality of care, and they can also be used to test whether an equitable service is offered to the different socioeconomic strata in the population. It is generally recognized that health care inequalities exist, which may be classifiable using socioeconomic measures, or by ethnic group or gender [6], but it is important to consider whether such inequalities exist for different age groups too. Some studies, for example, have identified an “under-prescription” phenomenon for the chronically ill among the oldest old [7], and this also relates to cases of coronary heart disease (CHD) [8]. The differences identified could represent an excellent starting point for efforts to better define optimum care or best practices, to design care processes that meet patients’ needs [3], and to improve the quality of primary health care in terms of an equitable management of chronic diseases. With this in mind, it becomes essential to consider health system performance indicators also in terms of the different age groups comprising the population [9].

The aim of the present study conducted in Italy was to ascertain whether adherence to disease management guidelines is the same in all age groups of patients with diabetes, congestive heart failure (CHF) or CHD.

## Methods

Italy is divided administratively into 20 regions, and each regional government is responsible for fulfilling the objectives of the National Health Plan in its area. These regional authorities plan and organize health care facilities and activities through their regional health departments. They also coordinate and control local health units (LHU), each of which is a single National Health Service (NHS) unit that plans and delivers health care services to its local community. Each LHU is organized into geographical subareas called Health Districts (HD), which manage all the local primary health care structures and community services.

In Italy, all citizens are registered with a freely-chosen general practitioner (GP), and these GPs have a gate-keeping role. There are established drug prescriptions, exemptions from prescription charges, and diagnostic tests specific for a given chronic condition for patients with certain chronic diseases, as listed in a Decree of the Ministry of Health approved in 1999. To obtain drugs and diagnostic tests free of charge, patients need to exhibit their GP’s prescription.

### Data and Variables

Six Italian regions, two in northern Italy (Lombardy and Veneto), three in central Italy (Emilia Romagna, Tuscany and Marche), and one in southern Italy (Sicily) took part in the VALORE project, an initiative of the National Agency for Regional Health Systems for the purpose of assessing quality of care for chronic diseases and the organization of primary health care services [10]. The present study was an offshoot of this project. One or two LHUs from each region were involved (8 in all), and 2–4 HDs for each LHU (amounting to 21 in all), which shared their data (each regional authority independently chose which LHUs and HDs to enroll in the study). The dataset used in our analysis was generated by automatically processing administrative records. The following data files were used: a) hospital discharge records with one main and five secondary diagnoses coded using the International Classification of Diseases, Ninth Revision, Clinical Modification (ICD9CM); b) drug dispensing records coded using the Anatomical Therapeutic Chemical (ATC) codes for drug classification (the ATC system is the drug classification system adopted by the World Health Organization), excluding drugs administered in hospital; c) disease-specific exemptions from copayment for health care, coded using the ICD9CM; d) the population registry with demographic details (year of birth, gender); and e) the outpatient care database (recording visits to doctors, blood tests or other diagnostic examinations). In each region, record-linkage within and between data files was done deterministically using a unique, coded personal identifier. The number of individuals aged 16 years or more as at 1 January 2008 amounted to 1,948,622; the cases of diabetes, CHD or CHF were identified by means of algorithms developed by the Tuscany Regional Public Health Agency, based either on the diagnoses reported in the hospital discharge records or on disease-specific drug dispensing records or disease-specific health care copayment exemptions [11]. This procedure led us to identify 105,987 patients with diabetes, 86,725 with CHD, and 28,062 with CHF, who formed the initial sample of patients considered in this study.

Process indicators for the purpose of assessing what health care providers did for their patients and how well they did so [12] were chosen among those identified and defined by scientific associations as quality measures of interest for improving outcomes for outpatients. In particular, for the diabetics we measured three indicators that the OECD considers indicative of the quality of care for diabetes at health system level [13], i.e. annual HbA1c testing; annual screening for nephropathy; annual LDL cholesterol testing. These indicators were calculated in terms of: the percentage of patients who had one or more HbA1c test a year; the percentage of patients who had at least one micro-albuminuria test during the year considered; the percentage of patients who had at least one annual LDL cholesterol test.

For cases of CHD, we measured three indicators that the Chronic Stable Coronary Artery Disease Work Group [14] considers indicative of the quality of care for CHD with a view to improving outcomes for outpatients with chronic stable coronary artery disease, i.e. therapy with angiotensin-converting enzyme (ACE) inhibitors; therapy with anti-thrombotic agents; and annual total cholesterol monitoring. These indicators were calculated in terms of: the percentage of patients who had at least two prescriptions of ACE inhibitors in the same year, separated by an interval of at least 180 days; the percentage of patients with at least two prescriptions of anti-thrombotic agents, separated by an interval of at least 180 days; the percentage of patients with at least one total cholesterol test a year.

For patients with CHF, we chose four indicators that the Heart Failure Work Group [15] considers indicative of the quality of care for CHF in terms of improving outcomes for outpatients with heart failure, i.e. therapy with ACE inhibitors; therapy with beta-blockers; 6-monthly monitoring of creatinine, Na and K; and annual echocardiography. These indicators were calculated in terms of: the percentage of patients with at least two prescriptions of ACE inhibitors in a year, separated by an interval of at least 180 days; the percentage of patients with at least two prescriptions of beta-blockers, separated by an interval of at least 180 days; the percentage of patients with at least one creatinine, Na and K test in the previous six months; and the percentage of patients completing at least one echocardiogram a year. These indicators were computed during a one-year follow-up (1 January 2009 to 31 December 2009) by linking the three pathology cohorts of patients to the administrative databases recording prescriptions for drug dispensing and diagnostic tests. To overcome any selection bias that might undermine the validity of our results we excluded all patients lost to follow-up because this loss is associated in our database with both exposure (age group) and outcome (adherence to the process indicator). The analyses were performed on 102,207 diabetic patients, 81,542 CHD and 24,997 CHF patients.

We only enrolled people registered with the Italian NHS (all Italian citizens and regular immigrants, i.e. foreigners who have a regular entry visa or residence permit) and we classified nationality as follows: Italians; immigrants from highly-developed countries (HDC); and immigrants from high migratory pressure countries (HMPC) [16].

The Charlson index was calculated to assess patients’ comorbidities: this index has proved a valid and reliable method for measuring comorbidities for the purpose of clinical research and, although it was first developed and validated for hospitalized patients, it has since been adapted and validated for primary care and community populations too [17].

### Statistical Methods

The data were summarized as numbers (percentages) of subjects for categorical variables. The chi-square statistic was used to test the hypothesis of independence between age group and adherence to standards of care. A multilevel logistic regression model was applied to analyze the association between age group and compliance with standards of care. The data had a hierarchical structure, with the patient on the first level and the HD on the second level. The dependent variables were analyzed in dichotomous form (yes/no) for each patient’s compliance with evidence-based quality of care requirements for the management of the diseases considered. In addition to the independent dummy variable age group (reference age group 65–74 y), the covariates in the regression model were: gender, nationality, time since diagnosis (dichotomized as ≤3 y and >3 y), and Charlson index on the first level, and HD on the second. A multilevel logistic regression model was also applied, similar to the one previously described except that age group (16–44, 45–64, 65–74, 75–84, >85 years old) was included in the model as an independent categorical variable, and also a quadratic term was used for the age group variable (enabling us to verify the hypothesized U-shaped curve).

Individuals whose citizenship was not known were excluded from the regression analysis (this applied to 5.6% of the diabetics and CHD patients, and to 4.78% of the CHF patients), so the multilevel regression analyses were performed on 96,529 patients with diabetes, 76954 with CHD, and 23,805 with CHF.

Data were analyzed using STATA software version 12.

### Ethics Statement

The study complied with the Declaration of Helsinki and with Italian Law (Decree n. 196/2003) on the protection of personal data. Resolution n. 85/2012 of the Guarantor for the protection of personal data also recently confirmed that it is allowable to process personal data for medical, biomedical and epidemiological research purposes, and data concerning health status can be used in aggregate form in scientific studies [18]. No identifiable human data were used for this study. The dataset used in the study is not publicly available. Permission to use non-identifiable individual data extracted from administrative databases for the VALORE project was granted by the ULSS 16 Padova, the ASP 7 Ragusa, the Assessorato Politiche per la Salute Emilia Romagna, the Zona Territoriale Senigallia, the Regione Lombardia, and the Agenzia Regionale di Sanità della Toscana, which are responsible for any use of the data concerning their respective populations. A disclosure statement was also submitted to the ethics committees of the Local Health Units in the areas participating in the study.

Approval for the use of encrypted and aggregated data was also obtained from the Italian College of General Practitioners.

## Results

The characteristics of the sample are shown in Table 1.

**Table 1:** Sample characteristics.

		CHD % (n)	CHF % (n)	DIABETES % (n)
**Sex**	**Male**	56.7% (46,255)	49.6% (12,394)	51.6% (52,760)
**Age group**	**16–44 yrs**	1.2% (947)	1.2% (303)	5.2% (5,332)
	**45–64 yrs**	16.6% (13,570)	11.1% (2,765)	27.6% (28,182)
	**65–74 yrs**	24.8% (20,201)	19.6% (4,891)	29.9% (30,586)
	**75–84 yrs**	35.6% (29,989)	37.8% (9,442)	27.4% (27,988)
	**85+ yrs**	21.9% (17,835)	30.4% (7,596)	9.9% (10,119)
**Citizenship**	**Italian**	98.8% (76,056)	98.9% (23,544)	96.9% (93,550)
	**HDC**	0.2% (186)	0.3% (62)	0.3% (283)
	**HMPC**	0.9% (714)	0.8% (199)	2.8% (2,696)
**Charlson index**	**no comorbidity**	56.7% (46,218)	26,8% (6,705)	68.6% (70,080)
	**low comorbidity**	19.6% (15,994	25.1% (6,267)	13.5% (13,829)
	**high comorbidity**	23.7% (19,330)	48.1% (12,025)	17.9% (18,298)
**Time since diagnosis**	**more than 3 yrs**	63.1% (51,467)	42.4% (10,592)	66.0% (67,461)

Table 2 shows details of the specific indicators monitored and highlights the differences by age group. For almost all indicators, the percentage of patients undergoing the recommended tests or taking the evidence-based recommended therapy was lower in the extreme age groups (i.e. the young adults and the oldest old) than in the middle age group (65- to 74-year-olds).

**Table 2:** Bivariate analysis: compliance with disease management standards by age group.

		16–44	45–64	65–74	75–84	85+	
		(n = 947)	(n = 13,570)	(n = 20,201)	(n = 28,989)	(n = 17,835)	p
**CHD1**	**ACE-inhibitor** **therapy**	28.9% (266)	56.8% (7,713)	65.0% (13,135)	63.4% (18,387)	52.8% (9,409)	<0.001
**(n = 8,542)**	**Total cholesterol**	36.6% (347)	58.5% (7,942)	65.1% (13,145)	59.6% (17,278)	40.4% (7,208)	<0.001
	**Anti-thrombotic** **therapy**	34.4% (326)	65.2% (8,844)	69.5% (14,047)	67.0% (19,408)	57.6% (10,280)	<0.001
		**(n = 303)**	**(n = 2,756)**	**(n = 4,891)**	**(n = 9,442)**	**(n = 7,596)**	**p**
**CHF**	**ACE-inhibitor therapy**	36.3% (110)	64.7% (1,788)	68.8% (3,363)	64.6% (6,099)	51.4% (3,904)	<0.001
**(n = 24,997)**	**β-blocker therapy**	38.3% (116)	55.7% (1,541)	51.3% (2,510)	41.6% (3,924)	26.5% (2,016)	<0.001
	**Creatinine, sodium,** **potassium tests**	42.9% (130)	48.5% (1,340)	57.7% (2,821)	60.7% (5,731)	55.8% (4,236)	<0.001
	**Echocardiography**	24.4% (74)	25.2% (698)	24.0% (1,174)	17.8% (1,682)	7.5% (569)	<0.001
		**(n = 5,332)**	**(n = 28,182)**	**(n = 30,586)**	**(n = 27,988)**	**(n = 10,119)**	**p**
**DIABETES**	**Creatinine** **measurement**	48.4% (2,580)	57.2% (16,108)	66.7% (20,413)	69.5% (19,457)	63.6% (6,440)	<0.001
**(n = 102,207)**	**HbA1**	48.1% (2,562)	60.9% (17,164)	66.8% (20,431)	63.0% (17,628)	50.0% (5,045)	<0.001
	**Lipids**	46.4% (2,475)	58.7% (16,530)	64.5% (19,723)	59.7% (16,715)	43.5% (4,400)	<0.001

**Figure 1:**
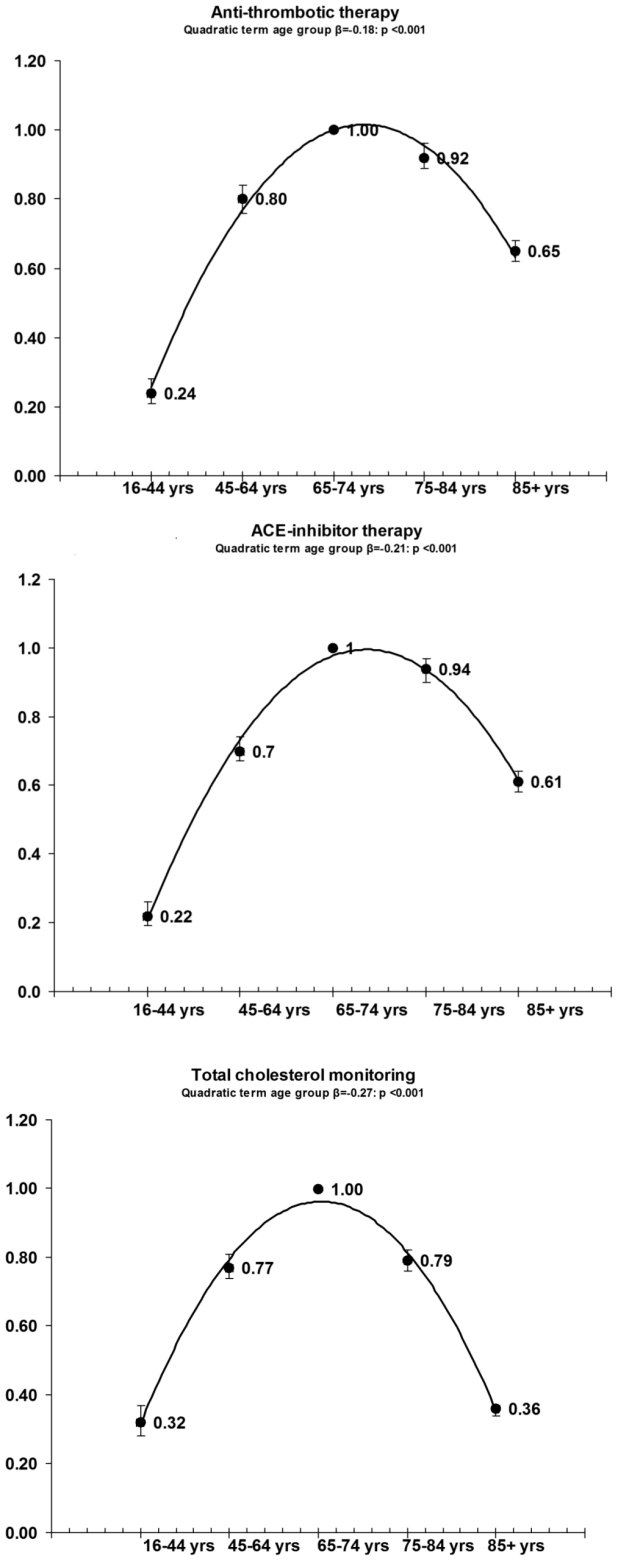
OR and 95%CI of multilevel logistic adjusted regressions in CHD patients: dependent variable = compliance with disease management standards; independent variable = age group.

**Figure 2:**
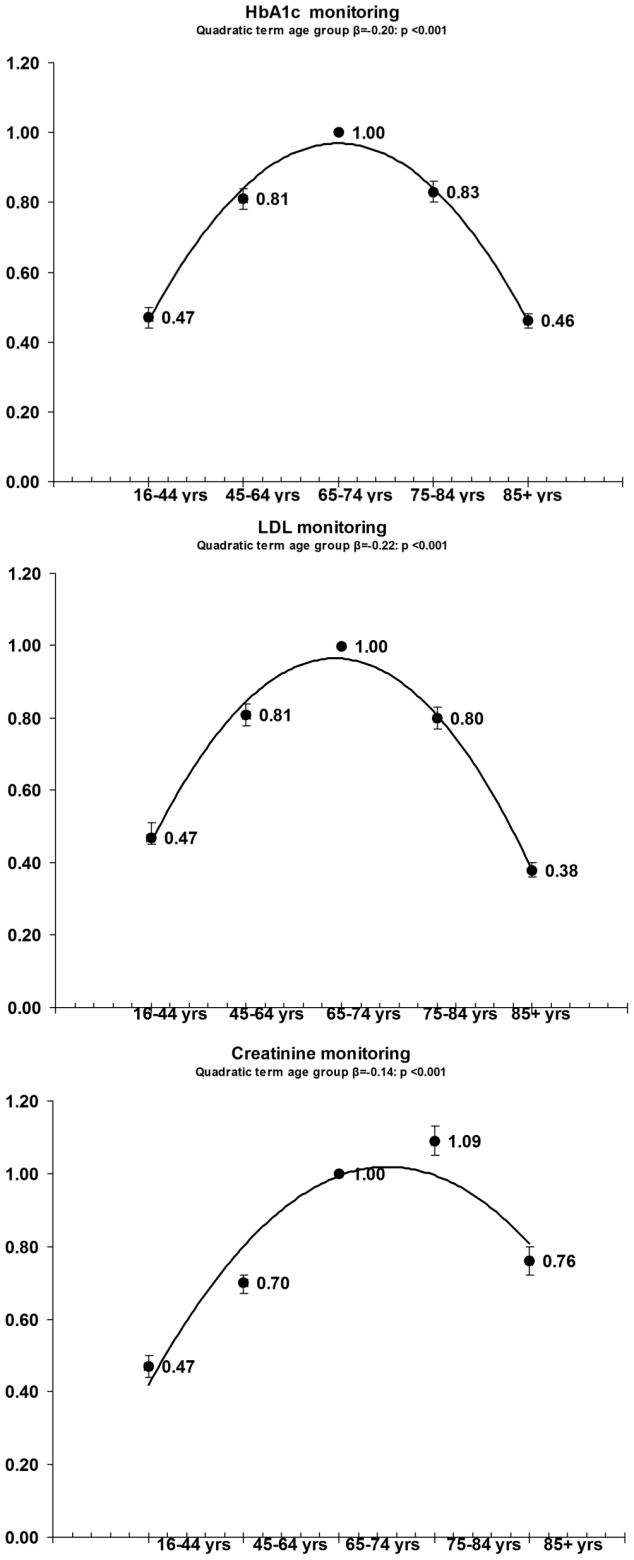
OR and 95%CI of multilevel logistic adjusted regressions in diabetic patients: dependent variable = compliance with disease management standards; independent variable = age group.

**Figure 3:**
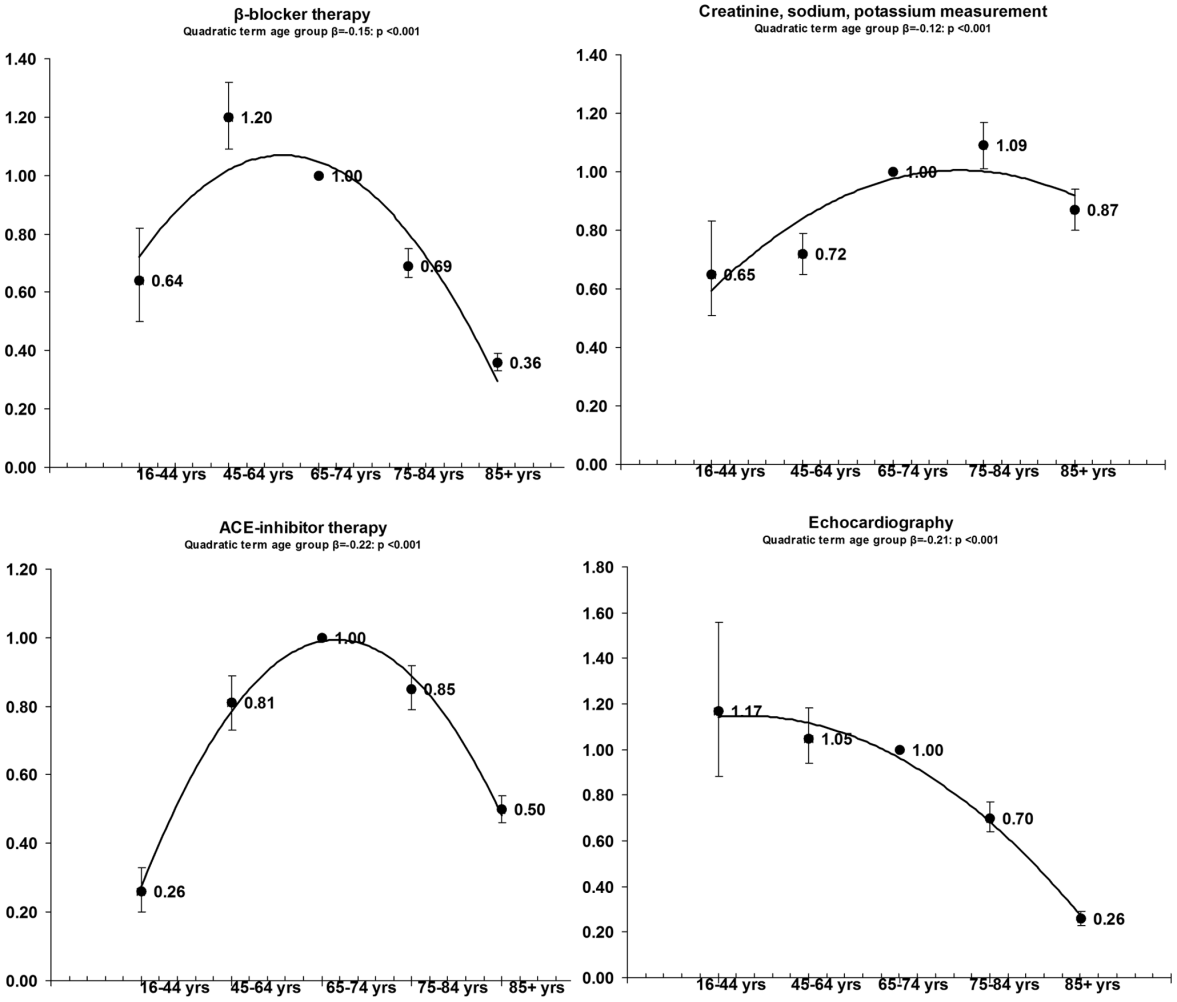
OR and 95%CI of multilevel adjusted logistic regressions in CHF patients: dependent variable = compliance with disease management standards; independent variable = age group.

The results of our multilevel logistic regressions (Figures 1, 2, 3) systematically confirmed age-related differences in all quality management indicators for the three chronic diseases considered, showing an inverted U-shaped relationship with age in almost all process indicators. Compliance with the recommended guidelines was greater in the young elderly age group than for any of the other age groups for almost all the indicators relating to all three chronic diseases studied. By comparison with the young elderly (65- to 74-year-olds), young adults (16- to 44-year-olds) with diabetes had more than 50% lower odds of all three quality indicators (lipids, HbA1c and creatinine) being monitored annually; young adults with CHD had a more than 70% lower likelihood of being treated with ACE inhibitors and anti-thrombotic agents, and almost 70% lower odds of having their cholesterol profile checked; and young adult CHF patients had more than 70% lower odds of being treated with ACE inhibitors, as well as more than 30% lower odds of being treated with beta-blockers and having their creatinine, sodium and potassium levels monitored annually.

## Discussion

To the best of our knowledge, this is the first study to be conducted in Europe to assess the equity of access to health services for chronic diseases by age group. Age-related disparities systematically emerged in patients’ compliance with the recommended guidelines for the care of diabetes, coronary artery disease and congestive heart failure, generating an inverted U-shaped relationship with age in terms of patients’ compliance with standards considered for these chronic conditions. In particular, our results indicate that young adults (16- to 44-year-olds), middle-aged adults (45- to 64-year-olds), the very old (75- to 84-year-olds), and the oldest old (85 and over) with CHD, CHF or diabetes are less likely to be monitored and treated according to the evidence-based recommendations than patients aged 65 to 74, with the sole exceptions of echocardiographic monitoring in young adult CHF patients and renal monitoring for very old CHF and diabetic patients. These results are alarming because an inadequate management of chronic conditions means a worse outcome for patients and consequently higher health care costs [19].

These findings indicate that, while the Italian population is increasingly liable to chronic conditions, the health care delivery system has hitherto remained poorly organized to provide care for these patients. The picture is much the same elsewhere around the world, where it has been reported that health care systems are not well designed to meet the needs of the chronically ill [3]. The current health care delivery system responds primarily to acute and urgent health problems, with the emphasis on diagnosing a patient’s condition, ruling out serious diseases, and relieving symptoms. Patients with chronic conditions would be better served by a systematic approach focusing on self-management, with a multidisciplinary team planning their care, routine assessment and follow-up [20]. There are possibly two main reasons for our results, explaining why the health care delivery system succeeded in managing the older adult (but not the very old) patients more systematically: one is that patients in this age group more frequently seek primary health care spontaneously; the other is that these patients are more likely to adhere to their primary care physicians’ recommendations for their treatment and monitoring. The situation could also have to do with the Italian primary health care model being based on a “waiting paradigm”, meaning that an event has to occur before action is taken to solve the problem. Waiting is the classical health care paradigm of the biomedical model, and it has also become the dominant paradigm in territorial and primary health care [21]. Patients aged 65 to 74 could access health care services more easily because they are more likely to be retired than younger adults, and more likely to be able to move unassisted than older people, and therefore go to see physicians, who “wait” for patients to come to them. Wagner et al [20] made the point that successful chronic disease management programs should: (i) produce protocols or plans that state explicitly what needs to be done for patients, at what intervals, and by whom, and that considers the needs of all patients with specific clinical features, and how their needs can be met; and (ii) use registries to inform health care providers about which patients have certain conditions and thereby enable these patients’ proactive clinical management. Using reminder systems improves patients’ participation in appropriate care plans. With this in mind, one way to reduce age-related disparities in the management of chronic conditions would be to adopt a more proactive approach to primary health care, seeking to identify patients’ needs without waiting for them to come forward [5]. Such a model had not been applied in Italy up until the period generating the data analyzed in the present study, but it has since been adopted in some Italian regions, based on an integrated chronic care model [22], [23]. Further research will be useful to see whether this can reduce age-related inequalities in chronic disease management.

The results of our study are partly concordant with a previous Canadian study in which older adults reported receiving better health services for chronic disease management than the very elderly [24]. This study assessed the family physician’s intent by measuring prescriptions or recommendations, so the results cannot reflect the patient’s compliance. Our study focused instead on compliance with disease management guidelines, assessed by means of an administrative database, so our findings could be associated not only with the equity of care provided by physicians (as in the Canadian study), but also with patient compliance. Adherence is a multifaceted behavioral issue influenced by how health care is delivered by health care providers’ practices, as well as by patient-related factors [25]. Age has an important influence on non-compliance with health professionals’ recommendations, and different age groups have a different health-related behavior, more pertinent to their needs, the amount of time they have available, and so on [26]. Adherence to therapy and monitoring is crucial to preventing complications in the chronically ill and, by adopting a collaborative care model for chronic illness, health care providers can promote their patients’ self-management with a view to improving the chances of their disease being handled effectively by helping patients and families to cope with self-care tasks [27]. To be most effective, health care providers should have a patient-centered approach, cultivate a collaborative relationship, communicate clearly, provide advice when patients are ready to hear it, and learn more about any new recommendations [28]. Several specific strategies can help patients to change their behavior. Effective ways to reduce their resistance include: emphasizing their personal choice and control; reassessing their readiness, their conviction of the importance of their actions, and their confidence in the outcome; and sometimes backing off and supporting patients’ own decisions [29], or supporting patients capable of going online to obtain test results, take part in interactive care management services, and receive after-care instructions - by means of appropriately structured e-mail communications between patients with the same condition, for example [3]. Wagner et al [20] emphasized that a successful disease management program should include a strong focus on patient information and self-management (so that patients and their families acquire the skills they need for their self-management) and effective communications, both among caregivers and between caregivers and patients. Disease management programs (DMPs) can improve health: they have proved successful in increasing health care providers’ compliance with guidelines [30], and in improving patients’ disease control in conditions such as diabetes [31], [32]. The economic efficacy of DMPs is unclear, however [33]. Some studies have reported net cost savings after adopting DMPs, and a return on investment of $1.26 per $1.00 spent on disease management services for patients with congestive heart failure and diabetes [34].

The strength of our study lies in that it was conducted on an unrestricted and unselected population. Our study has some limitations too, however. First of all, not all the relevant socio-economic factors were available in the database. One example concerns the level of formal education, which could be a confounding factor. On the other hand, the association between age and education is unlikely to explain the reported U-shaped trend of the association between age and outcome, since age and education are linearly related [35]. These data could be biased, however, due to an opportunistic sample of LHUs being enrolled by the regional systems. There is also the risk of administrative databases overestimating prevalence because of either a lack of specificity of the case ascertainment algorithm or a failure to differentiate co-morbidity overlaps in prescriptions. These important methodological issues were addressed by a recent paper demonstrating the consistency of the Valore database [11] - used in the present study - with other sources of data, such as primary care medical records and national surveys. Another limitation of studies that make a secondary use of existing healthcare data sources could lie in that they are based only on the prevalence of diagnosed cases, from which the actual prevalence in the population cannot be estimated [31]. Having said that, cases that have yet to be diagnosed cannot be monitored, so this limitation does not affect our results in terms of estimating the association between monitoring practice and age group.

Finally, all drug-dispensing services and diagnostic or follow-up tests funded by the national health system are recorded in our database. Drugs purchased out-of-the counter or diagnostic and follow-up tests paid out-of-pocket are not recorded, but there is no evidence that use of such services is unevenly distributed across age bands. On the other hand, the NHS database does not record drug prescriptions for older people in long-term residential care, because rest homes stock drugs without any reference to the individual patients requiring them. The measurement bias due to this issue was assessed by taking into account the number of elderly people (over 65 years old) in long-term residential care in the Regions considered in this study according to the National Agency for Regional Health Services data [36], weighted with the number of chronic patients cases obtained by each region. This analysis generated an estimated 4‰ data misclassification approximately for drug prescriptions for the elderly age group. Such a bias however could not affect the indicators relating to diagnostic and instrumental tests because such prescriptions are always recorded at the individual level.

In conclusion, creative solutions are needed to address the escalating health care burden of chronic diseases. All chronic conditions place heavy demands on health systems, and comparable ways of organizing health care are similarly effective regardless of the biomedical etiology involved [3]. Adopting evidence-based approaches can make health care systems more coherent and efficient, and provide a means for improving quality across a range of chronic health problems, as well as ensuring that primary health care really is a service that comes as close as possible to where people live and work, with a level of care that ensures fewer health disparities across population subgroups (including those related to age) [37]. More action is still needed to promote a proactive, integrated approach to chronic care capable of involving chronic patients in all the seasons of their life.
